# Remnants of the Past or Ready to Move? Resident Birds Display Migratory Restlessness

**DOI:** 10.1371/journal.pbio.0040130

**Published:** 2006-04-04

**Authors:** Liza Gross

In a remarkable display of endurance and fitness, arctic terns fly up to 20,000 miles between their Arctic breeding grounds to the Antarctic seas each year. But most long-distance fliers rack up considerably less mileage, and rely on extra fat storage rather than snacking along the way, as terns do. Still other migrating birds travel just a few miles between alpine meadows and lowlands to find optimal food and shelter. Some fly at night, others during the day; some over land, others over water. No one can say for sure how migration came about, but climate, competition for resources, and the availability of food all likely played some role in this ancient behavior.

Studies of migratory behavior have shown that captive migratory birds demonstrate a seasonally appropriate spontaneous urge to migrate, called Zugunruhe (pronounced zook-oon-roo-ha). This behavior varies with the species studied, with amount and direction of activity reflecting the species' natural migratory distance and route, suggesting that the migratory urge is innate. In a new study, Barbara Helm and Eberhard Gwinner took a different approach to studying migratory behavior. Rather than focusing on a migrating species, they decided to investigate the possibility that resident species also bear elements of Zugunruhe—and discover that “a readiness to move is common in birds.”

Helm and Gwinner searched for signs of migratory behavior in two subspecies of stonechats, *Saxicola torquata*, comparing a migrant that breeds in Austria, *S. t. rubicola*, and its equatorial resident relative, *S. t. axillaris*. European stonechats are short-distance, nocturnal migrators—they winter around the Mediterranean Sea—that begin their journey when daylight lasts just over 12 hours. Since they would otherwise be sleeping at night, nocturnal activity can serve as a proxy for Zugunruhe. African stonechats are sedentary species that do not abandon their breeding grounds in Kenya. Since the genetic and evolutionary divergence between stonechat taxa is large (these two subspecies diverged between 1 million and 3 million years ago), it's reasonable to predict that African stonechats would neither possess an internal migratory program nor display migratory restlessness. On the other hand, the evidence that migratory birds adjust their flight patterns in response to environmental changes and the suggestive evidence that resident birds display traces of migratory restlessness raises the possibility that migration may not be an all-or-nothing trait.

To investigate the presence of Zugunruhe in a resident species, the researchers raised and bred the offspring of Kenyan stonechats in their lab in Germany. One group of these birds was held for the duration of a migratory period under the nearly equal light and dark conditions of their native habitat, and a subset remained under these conditions for a year and a half. A control group was exposed to the natural seasonal light fluctuations of southern Germany. Helm and Gwinner recorded the birds' nocturnal movements with infrared motion sensors, and counted the number of movements within ten-minute intervals. If 20 or more movements were noted, the interval was considered “active.”

Even though the African stonechats experienced no temporal cues—light levels remained constant—their nocturnal activity roughly tracked the season. The African birds' migratory restlessness, marked by repeated, spontaneous outbursts of nocturnal activity, echoed that seen in European stonechats, though it was less pronounced. The African birds also showed a telling relationship between hatching date and onset of nocturnal activity: just like their migratory counterparts, late-hatching birds became restless earlier and earlier, coinciding with the migratory season.

The African birds' behavior can be attributed only to Zugunruhe, the researchers concluded, suggesting the influence of an inborn, precisely timed migratory program. The presence of this program in both migrants and residents suggests that the urge to migrate may have evolved in their common ancestor.

It's not clear what mechanism preserved the trait in the residents. It could be adaptive: Southern African stonechats, it's thought, migrate short distances up and down mountains, so it's possible that drought or other seasonal conditions could force the Kenyan birds to periodically take wing as well. Alternately, stabilizing selection may have protected the trait from extreme variation, or it may support the dispersal of young birds to new territories.

Whatever forces have retained this trait, Helm and Gwinner propose that it may be a common avian feature. Given the proper environmental triggers, this innate migratory program might kick in to allow birds to escape deteriorating habitats caused by global climate changes or other ecological disturbances. With evidence that Zugunruhe exists in nonmigratory birds, researchers can continue exploring migratory behavior in any number of resident-migratory pairs to probe the many ways birds take flight to improve their chances of survival.

## 

**Figure:**
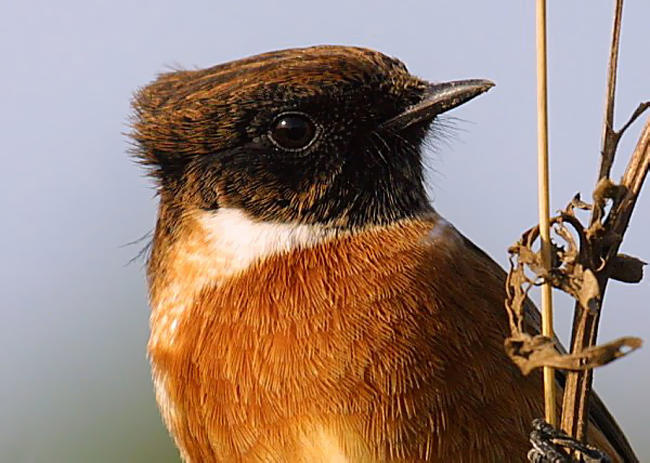
Recording migratory restlessness in resident African stonechats (the European stonechat is pictured above) suggests that nonmigratory birds retain an innate program supporting a seasonal urge to migrate.

